# Insulin-Like Peptides and Cross-Talk With Other Factors in the Regulation of Insect Metabolism

**DOI:** 10.3389/fphys.2021.701203

**Published:** 2021-06-29

**Authors:** Szymon Chowański, Karolina Walkowiak-Nowicka, Magdalena Winkiel, Pawel Marciniak, Arkadiusz Urbański, Joanna Pacholska-Bogalska

**Affiliations:** ^1^Department of Animal Physiology and Development, Faculty of Biology, Adam Mickiewicz University, Poznań, Poland; ^2^HiProMine S.A., Robakowo, Poland

**Keywords:** insulin-like peptides (ILPs), insulin-like growth factors (ILGFs), neuropeptides, metabolism, insects, cross-talk

## Abstract

The insulin-like peptide (ILP) and insulin-like growth factor (IGF) signalling pathways play a crucial role in the regulation of metabolism, growth and development, fecundity, stress resistance, and lifespan. ILPs are encoded by multigene families that are expressed in nervous and non-nervous organs, including the midgut, salivary glands, and fat body, in a tissue- and stage-specific manner. Thus, more multidirectional and more complex control of insect metabolism can occur. ILPs are not the only factors that regulate metabolism. ILPs interact in many cross-talk interactions of different factors, for example, hormones (peptide and nonpeptide), neurotransmitters and growth factors. These interactions are observed at different levels, and three interactions appear to be the most prominent/significant: (1) coinfluence of ILPs and other factors on the same target cells, (2) influence of ILPs on synthesis/secretion of other factors regulating metabolism, and (3) regulation of activity of cells producing/secreting ILPs by various factors. For example, brain insulin-producing cells co-express sulfakinins (SKs), which are cholecystokinin-like peptides, another key regulator of metabolism, and express receptors for tachykinin-related peptides, the next peptide hormones involved in the control of metabolism. It was also shown that ILPs in *Drosophila melanogaster* can directly and indirectly regulate AKH. This review presents an overview of the regulatory role of insulin-like peptides in insect metabolism and how these factors interact with other players involved in its regulation.

## Introduction

**FIGURE 1:**
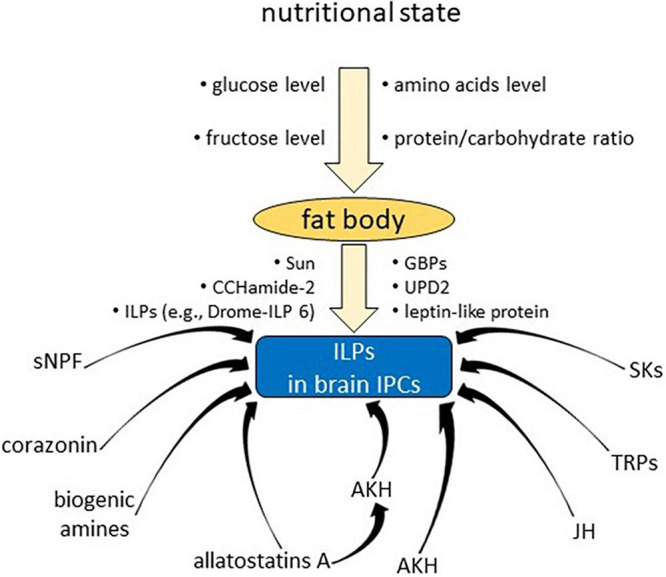
Examples of various factors affecting synthesis/secretion of insulin-like peptides in insulin producing cells in the brain.

**FIGURE 2:**
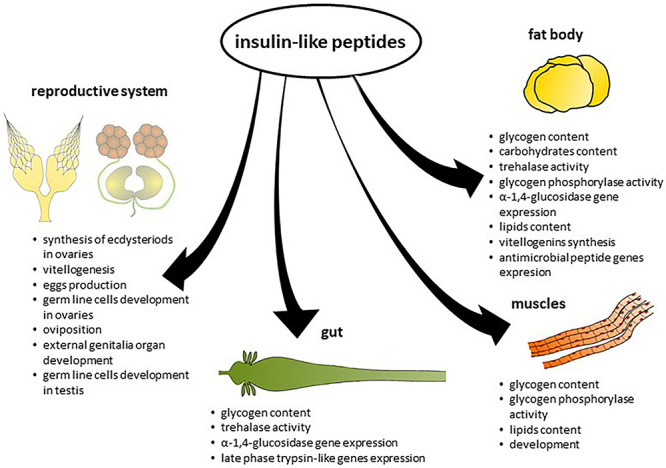
Effects of insulin-like peptides on different tissues.

The insulin/insulin-like peptide signalling (ILP signalling) pathway is an old and evolutionarily conserved pathway that widely regulates metabolism throughout the whole metazoan kingdom (Jin Chan and Steiner, 2000; Fernandez and Torres-Alemán, 2012; Vitali et al., 2018; Sharma et al., 2019). ILP signalling is involved in the control of metabolism *sensu stricte*, as well many other aspects of life, such as growth, reproduction, lifespan, resistance to stress conditions and immune activity. All these processes are directly or indirectly connected with metabolism. The best known ligand of ILP signalling pathways is mammalian insulin, probably the most deeply studied peptide hormone, but the family of insulin peptides is much larger (Veenstra, 2020). In humans, it also includes two insulin-like growth factors (IGFs), one relaxin and a number of human insulin-like peptides (INSL3-7) (Nässel and Vanden Broeck, 2016). They are synthesized as pre-propeptides consisting of a signal peptide and contiguous B-C-A peptides. The C-peptide is removed from insulin and relaxin, whereas IGFs contain a shortened C-peptide that is not excised. Thus, insulin and relaxin are heterodimeric peptides consisting of A- and B-chains linked by two to three disulphide bridges, and IGFs are single chain peptide hormones (Grönke and Partridge, 2010). In insects, the insulin peptide family is represented by numerous insulin-like peptides (ILPs) and IGF-like growth factor peptides (IGFLPs), but their number varies significantly between different species. For example, in *Locusta migratoria* and *Schistocerca gregaria*, only one ILP was identified, while there are five in *Anopheles gambiae*, eight in *Aedes aegypti* and *Drosophila melanogaster*, and 38 in *Bombyx mori* (Nässel and Vanden Broeck, 2016; Sharma et al., 2019). The target of insulin family peptides is insulin receptors (IRs). To date, several IRs belonging to the family of tyrosine kinase receptors or to G-protein coupled receptors have been identified in mammals. For a long time, only one receptor was identified in insects, but now, additional receptors have been identified, e.g., Lgr3 in *D. melanogaster* (Colombani et al., 2015; Van Hiel et al., 2015). The activation of IRs switches on a cascade of intracellular signalling reactions that trigger changes in cell activity. The multiplicity of ILPs, which possess various affinities to facilitate binding to different IRs, and the production and secretion of ILPs by different tissues result in signalling *via* ILPs that is not simple and straightforward. Signalling is more complex if it interplays with many other hormonal and nonhormonal factors, but in this way, regulation of metabolism and its coupled processes may occur with precision and in a multidirectional manner (Figures 1, 2).

In our work, we summarize knowledge how ILPs regulate the insect metabolism and known and possible interactions/cross-talk of ILPs with other agents controlling metabolic activity. In the following review, we used the nomenclature of peptide hormones according to Coast and Schooley (2011).

## Genes Encoding the ILP Family

In insects, two types of insulin peptide families have been identified: insulin-like peptides (ILPs) and IGF-like growth factor peptides (IGFLPs) (Mizoguchi and Okamoto, 2013; Okamoto and Yamanaka, 2015; Fujinaga et al., 2019). Most insect ILPs are classified in the first group. ILP genes encode precursors such as vertebrate pre-proinsulin, which contain signal peptide, the B-chain, C-chain, and A-chain, starting from the *N*-terminus (Antonova et al., 2012; Mizoguchi and Okamoto, 2013; Okamoto and Yamanaka, 2015). The signal peptide and *C*-peptide are cleaved, and the mature heterodimeric peptide consists of the A-chain and B-chain, which have approximately 20 and 30 amino acids respectively, linked by disulphide bonds such as in vertebrate insulin and relaxin (Mizoguchi and Okamoto, 2013; Okamoto and Yamanaka, 2015; Nässel and Vanden Broeck, 2016). In IGF-like peptides, a short C-chain is preserved, and the peptide is a single chain with internal disulphide bonds. The number and position of cysteine residues in ILPs and IGF-like peptides are highly conserved (Mizoguchi and Okamoto, 2013; Nässel and Vanden Broeck, 2016).

The three-dimensional structures of two insect ILPs have been determined so far, Bommo-ILP2 (called bombyxin — a name used for *B. mori* ILPs) (Nagata et al., 1995) and Drome-ILP5 (Sajid et al., 2011). The basic folding of Bommo-ILP2 and Drome-ILP5 molecules is similar to that in mammalian insulin, distinctions in conformation and function of their *C*-end of the B-chains were revealed (Nagata et al., 1995; Sajid et al., 2011). It was also detected that that Drome-ILP5 has features close to mammalian insulins, as it binds and activates human IR and decreases glucose level in rats (Sajid et al., 2011).

In most insects, multiple genes encode ILPs. For example, in *B. mori*, approximately 38 ILP genes have been identified (Mizoguchi and Okamoto, 2013); in *D. melanogaster*, there are eight ILP genes (Nässel et al., 2015), as in the mosquito *Ae. aegypti* (Riehle et al., 2006), and four in *Tribolium castaneum* (Li et al., 2008), but only one ILP gene in locusts is known to date (Badisco et al., 2008; Antonova et al., 2012). In the *B. mori* genome, most Bommo-ILP genes are gathered in two sectors on chromosome 11 and unidentified chromosome and form gene pairs, triplets or single genes. The rest are situated singly on chromosomes 1, 9 and 11 (Aslam et al., 2011; Sharma et al., 2018). Most of these genes lack any introns; however, three of them were found to have introns and encode polypeptides similar to pre-proinsulin, while several other genes are probably pseudogenes (Aslam et al., 2011; Mizoguchi and Okamoto, 2013). In *Ae. aegypti*, seven of eight Aedae-ILP genes have a single intron within the C-peptide, ranging from 53 bp to at least 286000 bp. One Aedae-ILP (Aedae-ILP6) has two introns, one in the signal peptide and one in the A peptide extension (Riehle et al., 2006).

The proximity of some ILP genes in mosquitoes suggests that they form a eukaryotic operon regulated by a single promoter. In *Ae. aegypti*, transcription of three genes occurs in the order Aedae-ILP8-ILP1-ILP3, and all have putative poly-A-sites, suggesting that these genes are transcribed simultaneously to form polycistronic pre-mRNA, which is next processed and capped to form monocistronic mature mRNAs. It was also suggested that the ILP operon exists in *A. gambiae* (Riehle et al., 2006).

## ILP Receptors

The ILP signalling pathway is initiated by the binding of ILPs to insulin receptors (IRs). IRs are part of the tyrosine kinase (RTK) receptor family, which contains enzymatic tyrosine kinase domains within their cytoplasmic part (Defferrari et al., 2018). In vertebrates, different distinct receptors in the IR subfamily can be distinguished (Hernández-Sánchez et al., 2008). These are the IR activated by insulin, the type 1 IGF receptor (IGF1R) activated by IGF1 or IGF2 and the type 2 IGF receptor (IGF2R) activated by IGF2. The IGF2R, as it is distinct from the others, belongs to the G protein coupled receptors (GPCRs) (Fernandez and Torres-Alemán, 2012). In invertebrates, including insects, mainly one or two IRs from the tyrosine kinase family (Defferrari et al., 2018), and two relaxin-like (LGR type C) GPCRs have been identified (Van Hiel et al., 2015). Single IRs were discovered in flies (Grönke and Partridge, 2010; Xu et al., 2015), mosquitoes (Nuss and Brown, 2018; Nuss et al., 2018), dung beetles (Lavine et al., 2013), moths (Fullbright et al., 1997), cockroaches (Abrisqueta et al., 2014) and kissing bugs (Defferrari et al., 2018), whereas two IRs were reported in honey bees (de Azevedo and Hartfelder, 2008), fire ants (Lu and Pietrantonio, 2011), brown plant hoppers (Xu et al., 2015), tenebrionid beetles (Sang et al., 2016), and aphids (Ding et al., 2017). If two receptors are present, they probably act *via* distinct regulatory pathways (Sang et al., 2016).

As a result of ligand stimulation, IR undergoes tyrosine phosphorylation. The IR precursor encodes A and B subunits that are connected by disulphide bridges and form a heterodimer. This dimer binds with a similar heterodimer, producing a mature and functional holoreceptor protein complex (Claeys et al., 2002). The A-chain and part of the *N*-terminus of the B-chain are located on the extracellular side of the plasma membrane. The remainder of the B-chain includes a single transmembrane helix, the juxtamembrane domain, and the intracellular tyrosine kinase domain. Individual subdomains that build the extracellular domain are characterized as leucine-rich, cysteine-rich and fibronectin type III domains (Scapin et al., 2018). Ligand binding specificity depends on cysteine-rich regions in the A subunits, whereas B subunits perform the tyrosine kinase activity mediating the ILP signal to downstream signalling proteins. Therefore, ILP binding is localized at the extracellular side, while interaction with downstream signalling factors occurs at the cytoplasmic side of the membrane receptor (Claeys et al., 2002). Among insects, the highest structural homology was observed in the tyrosine kinase domain (Defferrari et al., 2018).

Upon binding of insulin to its receptor, autophosphorylation of the intracellular IR subunits occurs due to the activation of their intrinsic tyrosine kinase activity. IR employs a group of adaptor molecules, known as insulin receptor substrates (IRSs), to initiate its signalling pathway (White, 1998). The interaction of IRS with the tyrosine-phosphorylated cytoplasmic tail of IR results in stimulation of the molecule by specific tyrosine residues phosphorylation in IRS molecule. Numerous tyrosine phosphorylation sites on IRS anchor molecules containing domains with Src-homology (SH2) (White, 1998). Two very important SH2-domain proteins can react with IRS, namely, Grb2 (growth factor receptor bound protein-2) and PI3K (phosphatidylinositol-3-OH kinase) (Claeys et al., 2002). Thus, IR stimulation typically activates two main intracellular pathways: the PI3K/Akt/FOXO (phosphatidylinositol-3 kinase/protein kinase B/Fork head box transcription factor) cascade and the Ras/MAPK pathway (mitogen-activated protein kinase) (Defferrari et al., 2018). The PI3K/Akt pathway regulates processes involving carbohydrates (mainly glucose) uptake and metabolism, whereas the IR-dependent MAPK pathway regulates mitogenic and cell cycle responses and is involved in control of development and reproduction (Sang et al., 2016).

The analysis of receptor or receptor expression profiles showed that in most insect species, IR is present in almost all tissues, with the highest expression level in the central nervous system and ovaries of different insect species (Defferrari et al., 2018). Moreover, in *D. melanogaster*, the receptor was found in imaginal discs and embryos (Garofalo and Rosen, 1988).

Developmental expression analysis showed that the highest expression of IRs was observed in adults; however, in all developmental stages (egg, larva, and pupa), a differentiated expression pattern was observed (Xu et al., 2015; Sang et al., 2016). In insects with two ILPRs, it was shown that both receptors might have different physiological significance (Lu and Pietrantonio, 2011; Sang et al., 2016) and that they functionally diverged between social and non-social insects (Sang et al., 2016).

G protein coupled receptors involved in ILP signalling thus far have been found only in *D. melanogaster* (Vallejo et al., 2015; Van Hiel et al., 2015). They belong to the GPCR subgroup, which contains leucine-rich repeats (LGRs), and are designated as type C (Van Hiel et al., 2015). This type of receptor also contains a very large *N*-terminal extracellular domain with multiple leucine-rich repeat motifs (LRRs) flanked by a cysteine-rich region and low-density lipoprotein receptor domain class A (LDLa), which are important for cyclic AMP signalling (Van Hiel et al., 2015). These receptors displayed high expression during development with higher expression levels in adult males (Van Hiel et al., 2015).

## Cells Producing ILPs

Insect ILPs are mainly considered neurohormonal agents regulating life processes; thus, their expression, synthesis and secretion are studied in the context of the nervous system. Nevertheless, their expression is widespread within whole organisms. The nervous system was the first tissue where insulin-like peptide-producing cells (IPCs) were identified. Mizoguchi et al. (1987) found four pairs of large mid-dorsal neurosecretory cells of the brain and nerve fibres located peripherally to the *corpora allata* (CA) in *B. mori*, which was confirmed by Ichikawa (1991). These cells were medial neurosecretory cells (MNCs) with axons that terminated in the CA (Mizoguchi and Okamoto, 2013). In *B. mori*, ILPs are synthesized mainly in the brain but they are produced also in numerous other tissues at low level (Iwami et al., 1996). However, only in brain, ILP genes are expressed during whole *B. mori* development from the embryonic to adult stages. ILPs were also detected in the ganglia, epidermis, testis, ovary, fat body, silk gland, Malpighian tubule, midgut, and hindgut but with different stage- and age-dependent patterns of ILP gene expression (Iwami, 2000). A similar situation occurs in *D. melanogaster*, where ILPs are synthetized mainly by the group of MSCs in the *pars intercerebralis* of the brain (Nässel, 2012; Nässel and Vanden Broeck, 2016), and the secretion of ILPs occurs from axons terminated in neurohaemal areas in the *corpora cardiaca* (CC), anterior aorta, and foregut (Brogiolo et al., 2001; Cao and Brown, 2001; Rulifson et al., 2002). However, as suggested by Nässel (2012), probably, Drome-ILPs might be secreted into the circulation from neurohaemal sites as well as in a paracrine way from branches within the brain. The crucial role of MSCs in ILP signalling in metabolism regulation has been shown by genetic cell ablation experiments (Rulifson et al., 2002; Broughton et al., 2005). Ablated larvae showed increased haemolymph sugar levels (Rulifson et al., 2002) and increased storage of lipids and carbohydrates (Broughton et al., 2005). In some insect species, other brain cells — lateral neurosecretory cells — are also involved in ILP production, for example, in *Ae. aegypti* mosquitos (Cao and Brown, 2001; Riehle et al., 2006) and *Anopheles stepensi mosquios* (Marquez et al., 2011) and in the hemipteran bug *Rhodnius prolixus* (Vafopoulou and Steel, 2012). On the other hand, IGF-like ILPs, such as Drome-ILP6 in *D. melanogaster* or Bomme-IGFLP in *B. mori*, are example of ILPS mainly synthetized by the fat body (Okamoto et al., 2009a, b; Mizoguchi and Okamoto, 2013). In Table 1, examples of ILPs expressed in different tissues/cells depending on the developmental stage are presented.

**TABLE 1:** Examples of ILPs expressed in different tissues/cells depending on the developmental stage.

**Insect species**	**ILP**	**Tissue presence of mRNA or protein**	**Developmental stage**	**References**
*Drosophila melanogaster*	Drome-ILP1	brain IPCs	larva; pupa	Brogiolo et al., 2001; Ikeya et al., 2002; Rulifson et al., 2002; Broughton et al., 2005; Veenstra et al., 2008; Yang et al., 2008; Géminard et al., 2009; Okamoto et al., 2009b; Garelli et al., 2012; Söderberg et al., 2012; Colombani et al., 2015; Nässel et al., 2015; Nässel and Vanden Broeck, 2016; Liu et al., 2016; Ohhara et al., 2018; Semaniuk et al., 2021b
	Drome-ILP2	brain IPCs	larva; adult	
		gut	larva	
		diuretic hormone 44-producing median neurosecretory cells	pharate adult	
		imaginal discs	larva	
		salivary glands	larva	
		mesoderm	embryo	
	Drome-ILP3	brain IPCs	adult; larva	
		midgut	adult	
		visceral muscles in the gut	adult; larva	
		mesoderm	embryo	
	Drome-ILP4	gut	larva	
		mesoderm	embryo	
		midgut	embryo	
	Drome-ILP5	brain IPCs	adult; larva	
		gut	larva	
		ovary	adult	
		Malpighian tubules	larva; adult	
		mesoderm	embryo	
	Drome-ILP6	fat body	larva; pupa; adult	
		gut	larva	
	Drome-ILP7	ventral nerve cord	larva	
	Drome-ILP8	imaginal discs	larva	
*Aedes aegypti*	Aedae-ILP1	brain	adult female	Riehle et al., 2006; Ling and Raikhel, 2021
		head	larva; pupa; adult male; adult female	
		thorax	larva	
	Aedae-ILP2	ovary	adult female	
		carcass (abdominal wall without fat body)	adult female	
		brain	adult female	
		Malpighian tube	adult female	
		midgut	adult female	
			embryo	
		head	larva; pupa; adult male; adult female	
		thorax	larva; pupa; adult male; adult female	
		abdomen	larva; pupa; adult male; adult female	
	Aedae-ILP3	brain	adult female	
			embryo	
		head	larva; pupa; adult male adult female	
		thorax	larva	
	Aedae-ILP4	brain	adult female	
		ovary	adult female	
		head	larva; pupa; adult female	
		abdomen	adult female	
	Aedae-ILP5	carcass	adult female	
		ovary	adult female	
		midgut	adult female	
		Brain	adult female	
		thorax	adult male; adult female	
		abdomen	larva; pupa; adult male; adult female	
	Aedae-ILP6	fat body	adult female	
		brain	adult female	
		midgut	adult female	
		thorax and abdomen walls without midgut	adult female	
			embryo	
		head	larva; pupa; adult male; adult female	
		thorax	larva; pupa; adult male; adult female	
		abdomen	larva; pupa; adult male; adult female	
	Aedae-ILP7	brain	adult female	
		ovary	adult female	
		head	larva; pupa; adult male; adult female	
		thorax	larva	
	Aedae-ILP8	brain	adult female	
		head	larva; pupa; adult male; adult female	
*Maruca vitrata*	Marvi-ILP1	brain	larva	Al Baki et al., 2019
		fat body	larva	
		midgut	larva	
		epidermis	larva	
		haemocytes	larva	
		head	adult male; adult female	
		thorax	adult male; adult female	
		abdomen	adult male; adult female	
			pupa	
	Marvi-ILP2	brain	larva	
		fat body	larva	
		midgut	larva	
		epidermis	larva	
		haemocytes	larva	
		head	adult male; adult female	
		thorax	adult male; adult female	
		abdomen	adult male; adult female	
			embryo	
			pupa	
*Blatella germanica*	Blage-ILP1	brain	adult female	Castro-Arnau et al., 2019
	Blage-ILP2	brain	adult female	
		ovary	adult female	
		fat body	adult female	
	Blage-ILP3	brain	adult female	
	Blage-ILP4	brain	adult female	
	Blage-ILP5	brain	adult female	
	Blage-ILP6	brain	adult female	
	Blage-ILP7	fat body	adult female	
*Nilaparvata lugens*	Nillu-ILP1	ovary	adult female	Lu et al., 2018; Xue et al., 2020
		head	adult female	
		fat body	adult female	
		midgut	adult female	
		epidermis	adult female	
			embryo	
			nymph	
	Nillu-ILP2	ovary	adult female	
		head	adult female	
		fat body	adult female	
		midgut	adult female	
		epidermis	adult female	
			embryo	
			nymph	
	Nillu-ILP3	fat body	adult female	
		ovary	adult female	
		head	adult female	
		midgut	adult female	
		epidermis	adult female	
			embryo	
			nymph	
	Nillu-lILP4	fat body	adult female	
			embryo nymph	
*Tribolium castaneum*	Trica-ILP1	brain	adult female	Sheng et al., 2011
		fat body	adult female	
	Trica-ILP2	brain	adult female	
		fat body	adult female	
	Trica-ILP3	brain	adult female	
		fat body	adult female	
	Trica-ILP4	brain	adult female	
		fat body	adult female	
*Spodoptera littoralis*	Spoli-ILP1	brain	larva	Van de Velde et al., 2007; Iga and Smagghe, 2011
	Spoli-ILP2	brain	larva	
*Spodoptera exigua*	Spoex-ILP1	fat body	larva	Kim and Hong, 2015
		epidermis	larva	
		head	larva	
		thorax	larva	
		abdomen	larva	
			embryo	
			pupa	
			adult	

## Factors Regulating ILP Production

Nutritional signals have been shown to be the most important factors that affect ILPs release from brain IPCs. It was demonstrated that Bommo-ILP is released from the brain of *B. mori* in response to availability of glucose, which is a widespread nutritional signal for releasing ILPs (Masumura et al., 2000; Mizoguchi and Okamoto, 2013). The Bommo-ILPs level in the brain decreased within 1 h after glucose injection into starved larvae of *B. mori* in a dose-dependent manner. It has also been demonstrated that the release of Drome-ILPs from IPCs of *D. melanogaster* is controlled by cell autonomous glucose sensing, comparable to mammalian pancreatic beta cells (Park et al., 2014). It was evidenced that in the glucose sensing of IPCs the glucose transporters, K_*ATP*_ channels and voltage-sensitive Ca^2+^ channels are involved as well (Fridell et al., 2009; Park et al., 2014; Nässel et al., 2015).

However, in *D. melanogaster*, the availability of nutrients is detected remotely by the fat body, which controls Drome-ILP releasing through humoral factors (Géminard et al., 2009; Bai et al., 2012; Rajan and Perrimon, 2012; Mizoguchi and Okamoto, 2013), and then amino acids instead of glucose become the crucial nutritional signal in the diet (Géminard et al., 2009). Amino acids do not directly impact the IPCs but rather they affect target of rapamycin (TOR) signalling pathway in fat body cells to regulate Drome-ILP release (Géminard et al., 2009). It was shown that amino acids deficit or inhibition of the TOR signalling pathway, both targeted in adipocytes, are enough to provoke Drome-ILP inhibition in IPCs and that Drome-ILP secretion is controlled by a direct humoral link between the fat body tissue and the brain (Géminard et al., 2009). It was also shown that changes in activation of the TOR signalling pathway in gut stem and/or progenitor cells caused the changes in Drome-ILP mRNA (Strilbytska et al., 2017a, b; Semaniuk et al., 2021b).

It has been detected in that *dilp* expression is affected also by the protein to carbohydrate ratio in the *Drosophila* diet and the interaction between this ratio and caloric content. For example, *dilp2* expression was the highest upon ingestion of diets with a low protein to carbohydrate ratio regardless of the total caloric value. *dilp5* expression increased at an approximately 1:2 protein to carbohydrate ratio and with caloric value of the diet (Post and Tatar, 2016; Semaniuk et al., 2021b).

Among detected nutrient signals that influence IPCs are unidentified factors discharged from the larval fat body cells in reaction to raised level of circulating amino acids (Géminard et al., 2009); leptin-like proteins secreted from the fat body after food intake in adults and affecting the GABAergic neurons in the brain (Rajan and Perrimon, 2012); and the brain cells that express a gustatory receptor (Gr43a) and respond to higher levels of fructose (Miyamoto and Amrein, 2014). Another fat body-derived diffusible molecule that controls the production and release of ILPs in adult *Drosophila* flies is Drome-ILP6. IGF-like Drome-ILP6 regulates carbohydrate and lipid storage, and its nutrient-dependent production is controlled by the FOXO transcription factor, which upregulated *dilp6* transcript level in the fat body (Slaidina et al., 2009; Bai et al., 2012). Drome-ILP6 produced by *Drosophila* fat body cells suppresses *dipl2* and *dilp5* mRNA in the brain, as well as the Drome-ILP2 release from IPCs (Bai et al., 2012). In adult flies, the activity of ICPs is regulated by the fat body cells not only *via* Drome-ILP6, but also by the leptin-like cytokine Unpaired 2 (Upd2) (Bai et al., 2012; Rajan and Perrimon, 2012). This probably occurs with mediation of the GABAergic system in the *pars intercerebralis*, which appears to be inactivated by circulating Upd2 after food intake; for that reason, tonic inhibition of the IPCs is raised (*via* activation of the Jak/Stat signalling pathway), which facilitates Drome-ILP secretion (Rajan and Perrimon, 2012). Besides, in response to dietary amino acids, two factors released from the fat body have been detected in *Drosophila*: Stunted (Sun) (Delanoue et al., 2016) and growth-blocking peptides (GBPs) (Koyama and Mirth, 2016). Sun is a circulating insulinotropic peptide released by adipose tissue and acts as a ligand for Methuselah (Mth), a secretin-incretin receptor subfamily member on IPCs, inducing the secretion of ILPs and promoting organ growth. GBPs are produced in the adipocytes in reaction to amino acids and activation of TOR signalling. GBPs stimulate Drome-ILP secretion from IPCs, which results in elevated ILP signalling activity in the body cells to promote body growth.

Furthermore, the results obtained by Alfa et al. (2015) showed the presence of an orphan GPCR in IPCs, which is a limnostatin receptor whose activation by limnostatin suppresses ILPs secretion from ICPs following starvation in *Drosophila*. The regulation of Drome-ILP2 and Drome-ILP5 synthesis by IPCs is probably also mediated by dSir2 (the *Drosophila* homologue of mammalian histone deacetylase SIRT1), but independent of the FOXO transcription factor (Kannan and Fridell, 2013), and by dCbl (Casitas B-lineage lymphoma), a member of the *Drosophila* E3 ubiquitin ligases and adaptor proteins, which downregulates the transcript level of brain *dilp* genes (Yu et al., 2012).

The ILPs expression and secretion undergoes regulation not only by ingested food but also, as was shown by Lushchak et al. (2015), by odor. These authors demonstrated that exposition of *D. melanogaster* to vinegar odor induces increased expression of *dilp2*, *dilp3* and *dilp5* in the brain IPCs. Moreover, they also observed increased expression of *dipl*6 and *upd2* (Lushchak et al., 2015).

Furthermore, it was proven that IPCs also receive regulatory signals from direct neuronal input as well as from hormonal factors (Figure 1; Antonova et al., 2012; Nässel and Vanden Broeck, 2016; Semaniuk et al., 2021b).

### Hormones

#### Corazonin and sNPF

In adult *Drosophila*, short neuropeptide F (sNPF) and corazonin (CRZ) are expressed in a bilateral set of neurons, the so-called dorsal lateral peptidergic neurons (DLPs), localized in the *pars lateralis*. These sNPF-expressing nervous cells have axon terminations impinging on IPCs (Kapan et al., 2012). IPCs express the sNPF receptor 1 (sNPFR1) and probably the corazonin receptor (Crz-R) (Kapan et al., 2012). It was suggested that sNPF secreted from DLPs targets IPCs to elevate production and most likely also the secretion of Drome-ILPs, since knockdown of *snpf*, but not *crz*, in DLPs decreased the levels of mRNA for *dilp2* and *dilp5* in the brain. It was also shown that knockdown of either *snpf* or *crz* in DLPs prolongs survival in starved flies and changes carbohydrate and lipid metabolism, which suggests that CRZ and sNPF act *via* different mechanisms (Lee et al., 2008; Kapan et al., 2012).

#### Tachykinin-Related Peptides

In *Drosophila*, receptors for tachykinin-related peptides (TRPs) are expressed by IPCs, and TRP knockdown significantly affects the mRNA levels of *dilp2* and *dilp3* but not *dilp5* in the brains of fed and exposed to starvation flies. *dilp2* and *dilp3* mRNA levels were elevated in fed flies, but in starved flies, *dilp2* was upregulated and *dilp3* was downregulated (Birse et al., 2011).

#### Allatostatin A

Another neuropeptide engaged in ILP signalling in *Drosophila* is allatostatin A (AstA). It was also shown that allotostatin A regulates AKH signalling. Expression of the AstA receptor gene *Dar2* was detected in the insulin- and AKH-producing cells (Hentze et al., 2015). Knockdown of *Dar2* in IPCs and AKH-producing cells (APCs) resulted in modification of expression of several genes that indicate decreased ILPs or AKH signalling. It was suggested that AstA regulates the balance between Drome-ILPs and AKH, which is believed thought to be essential to maintain the nutrient homeostasis in reaction to alternations of sugar and protein ratios in the diet (Hentze et al., 2015; Nässel and Vanden Broeck, 2016).

#### CCHamide-2

The CCHamide-2 (CCHa2) neuropeptide regulates IPCs and Drome-ILPs in a nutrition-dependent manner (Sano et al., 2015), and its transcription is altered, particularly in response to glucose levels. CCHa2 is produced primarily in the adipocytes and gut and directly stimulates its receptor (CCHa2R) in IPCs in the larval brain (Sano et al., 2015). In *D. melanogaster* mutants of both CCHa2 and CCHa2-R, the transcription of *dilp5* and the secretion of both Drome-ILP2 and Drome-ILP5 were reduced, and growth during larval stages was severely retarded (Sano et al., 2015).

#### Octopamine and Serotonin

In IPCs of *Drosophila*, the expression of two monoamine receptors, the octopamine receptor OAMB and the serotonin receptor 5-HT_1A_, has been detected (Luo et al., 2014). Knockdown of the OAMB receptor resulted in increased *dilp3* expression in the brain, whereas 5-HT_1A_ knockdown led to elevated transcript levels of *dilp2* and *dilp5* (Luo et al., 2014).

#### Dopamine

It was shown that dopamine stimulates its receptor DopR1 which is expressed in IPCs (Andreatta et al., 2018) and in female *D. melanogaster* promotes ovarian dormancy (Andreatta et al., 2018; Ahmad et al., 2020). Under normal, nondormancy conditions, Drome-ILP2 and Drome-ILP5 and juvenile hormone (JH) control ovarian growth and reproduction in females, and serotonin and dopamine signalling in IPCs, CA and fat body are diminished, and the dormancy is inhibited. In contrast, under dormancy-inducing conditions (e.g., low temperature), serotonin and dopamine restrain the production and/or release of Drome-ILPs in IPCs, triggering reduction of systemic ILP signalling (and JH signalling) and thus favouring a shift into the dormancy state (Andreatta et al., 2018).

#### Juvenile Hormone

Juvenile hormone and 20-hydroxyecdysone (20E) were shown to influence *ilp* gene expression in reproducing female *Ae. aegypti* mosquitoes. JH and 20E modulate the production and secretion of all eight Aedae-ILPs, restricting them to appropriate amounts required during the posteclosion and postblood-meal phases of the mosquito reproductive cycle. It was shown that the JH and 20E pathways act differentially in determining the expression of *ilp* genes. This is achieved through a direct physical interaction of JH and 20E pathway factors with promoters of *ilp* genes. *ilp2*, *ilp6* and *ilp7* are positively regulated by the JH pathway. In contrast, 20E pathway factors inhibit the expression of *ilp2* and *ilp6* genes directly interacting with their promoters. This situation is reversed in the regulation of *ilp4* and *ilp5* gene expression, in which genes are downregulated by the JH pathway factor and upregulated by the 20E pathway factor. It was also found that Met, a transcription factor identified as the JH receptor, provokes fat body *ilp6* expression by direct binding to this *ilp* gene promoter (Ling and Raikhel, 2021). Furthermore, JH elicits the expression of Trica-ILP2 and Trica-ILP3 in female *T. castaneum* and controls the expression of *vg* genes through the insulin pathway (Sheng et al., 2011). In *Apis mellifera*, JH works throughout Apime-ILP1 and controls metabolism of carbohydrates during the transition of worker bees from nursing to foraging (Wang et al., 2013). In *B. mori*, *in vitro* studies showed that secretion of the peptide Bomme-IGFLP from the fat body is stimulated by 20-hydroxyecdysone (20E), since both *bigflp* gene (encoding Bommo-IGFLP) expression and secretion of Bommo-IGFLP were significantly elevated by the supplementation of the adipocytes culture with 20E (Okamoto et al., 2009a). The expression of *dilp6* in the adipose tissue of *D. melanogaster* is also triggered *in vitro* by 20E (Okamoto et al., 2009a, b; Slaidina et al., 2009; Mizoguchi and Okamoto, 2013). However, Bomme-IGFLP expression in the brain was not elicited by 20E (Okamoto et al., 2011), which indicates that the mechanisms regulating ILP and IGFLP gene expression vary in different tissues. Starvation also caused upregulation of the *dilp6* expression in the larval adipocytes, through direct induction by the FOXO transcription factor, what was independent of 20E (Slaidina et al., 2009; Mizoguchi and Okamoto, 2013). Furthermore, FOXO-inducible *dilp6* expression was detected in the adult fat body (Bai et al., 2012).

### Other Factors

Other factors that have been demonstrated to control ILP expression in the brain and peripheral tissues in insects are microRNAs (miRNAs) expressed in IPCs. In *Ae. aegypti*, for lack of microRNA-277 (miR277), the mRNA levels of *ilp7* and *ilp8* were elevated in the head, while the mRNA levels of *ilp1* and *ilp3* transcript did not change, what suggests that miR277 targets the first member (*ilp8*) of the *ilp8-ilp1-ilp3* operon (Ling et al., 2017; Sharma et al., 2019). Genetic disruption of miR-277 led to impairment of lipid storage and development of ovaries development, suggesting that miR-277 acts as an essential factor in lipid metabolism and reproduction by targeting *ilp7* and *ilp8* and regulating the mRNA levels of these genes (Ling et al., 2017). In *D*. *melanogaster*, different microRNAs have also been found to control the production of Drome-ILPs in direct or indirect ways. miR-14 acts in IPCs in the adult *D. melanogaster* brain, targeting gene *sugarbabe*, which encodes a predicted zinc finger protein that controls *dilp* gene expression in IPCs. It was also shown that removing miR-14 reduces *dilp3*, *dilp5 and dilp2* transcript levels (Varghese et al., 2010). miR-9a has also been detected in *D. melanogaster* IPCs. Upregulation of miR-9a specifically in IPCs decreases ILP signalling and body size. miR-9a has been found to bind to *sNPFR1* mRNA in insect cells, suggesting its role in controlling body growth by regulating sNPFR1, which modulates ILP signalling (Suh et al., 2015). miR-278, expressed predominantly in the fat body, was shown to be involved in controlling of energy homeostasis in *D. melanogaster* by regulating insulin responsiveness (Teleman et al., 2006). Another fat body microRNA, miR-8, acts as a regulator of ILP-signalling *dilp6*, and *imaginal morphogenesis protein-late2* (*Imp-L2*), a Drome-ILP binding protein, was detected to be upregulated in the adipose tissue of miR-8 null mutant *Drosophila* larvae (Lee and Hyun, 2014).

## Regulation of Hormone Synthesis by ILPs

The participation of ILPs in the integration of metabolism and energy utilization also involves mediating the synthesis and release of various hormones with similar or antagonistic features (Nässel and Vanden Broeck, 2016). For example, ILPs are part of a complex relationship network that leads to the control of secretion from CA and adipocytes in fat body tissue *via* two insect hormones: JH and 20E (Nässel and Vanden Broeck, 2016). JH might be a product of direct (ILP may stimulate CA for JH synthesis) or indirect (synthesis of JH is under neuropeptide control, and ILPs might affect the neuropeptidogenic or somatic tissues) ILP activity (Tatar et al., 2003). On the other hand, ILPs might act directly on the ovary, where they take part in ovarian ecdysteroidogenesis (Tatar et al., 2001; Nässel and Vanden Broeck, 2016). ILPs are also suspected to engage in feedback with other neuropeptide pathways, such as SKs and NPFs (Wu et al., 2005; Lingo et al., 2007; Söderberg et al., 2012; Badisco et al., 2013). Furthermore, it is believed that together with AKH, ILPs work as counterparts of glucagon and insulin loops (Birse et al., 2011). ILPs stimulate carbohydrate uptake, which causes a reduction in trehalose levels in the haemolymph, while AKH increases trehalose levels by glycolysis stimulation but elicits little or no effect on glucose levels (Birse et al., 2011; Kim and Neufeld, 2015). Research shown that in *Drosophila*, *Akh* mRNA and AKH peptide are elevated in *dilp2* mutants although not in *dipl1* mutants or *dipl1-dilp2* double mutants, which suggests that *dilp2* epistatically downstream expression of *dilp1* what is required for *dilp2* to modulate AKH. Thus, it was proposed that Drome-ILP2 indirectly modulate AKH by reducing *dilp1* gene expression, while Drome-ILP1 otherwise activating AKH (Post et al., 2019). Notably, insulin-degrading enzyme (IDE) is also present in IPCs (Nässel and Vanden Broeck, 2016). IDE knockdown was shown to cause a reduction in carbohydrate levels in the haemolymph and increases fecundity and lifespan (Nässel and Vanden Broeck, 2016).

## Metabolic Processes Regulated by ILPs

### Food Intake

The neuroendocrine regulation of food intake in insects, as in other animals, is very complex (Nässel and Zandawala, 2019). It relies on the interplay of different neuropeptides, including ILPs (Nässel and Zandawala, 2020). The major neuropeptides acting as satiety and hunger signals are TKs, NPF, sNPF, SKs, AstA, hugin, leukokinins (LKs) and Upd1 (Nässel and Zandawala, 2020). The exact role of ILPs in this system is also quite complex and far from completely understood. Contrasting results regarding the role of ILPs in food intake regulation have been obtained. In general, ILPs have been shown to negatively regulate feeding and thus act as satiety signals in *D. melanogaster* (Söderberg et al., 2012; Pool and Scott, 2014; Semaniuk et al., 2021a). Semaniuk et al. (2021a) showed that *D. melanogaster* flies with knockout of different *dilps* ingested larger amount of carbohydrates. On the other hand, it was demonstrated that in *D. melanogaster* with the knockdown of insulin receptor in the progenitor and stem cells of the gut, the feeding activity was lowered as well as the glycogen and glucose content in the body (Strilbytska et al., 2020). Recent studies showed that also during starvation, blocking ILP signalling led to reduced feeding, whereas overexpression of ILP genes enhanced this process (Sudhakar et al., 2020). This shows that ILPs might be orexigenic during short periods of starvation and during extended starvation (Sudhakar et al., 2020). Moreover, upon feeding, satiety signals such as Drome-ILPs and other neuropeptides are released to terminate meal uptake (Nässel and Zandawala, 2020). Clearly, further detailed studies are needed to unravel the exact role of ILPs in this process.

In feeding regulation, ILPs interact with other neuroendocrine signals, such as SKs and sNPFs (Nässel and Zandawala, 2020). It was recently shown that ILPs form a positive feedback loop with sNPF during a short period of food deprivation. sNPF stimulates IPCs to produce ILPs, which in turn promote *snpf* gene expression (Sudhakar et al., 2020). sNPFs were previously shown to stimulate food intake (Fadda et al., 2019), in agreement with these studies. IPCs in *D. melanogaster* were shown to express, in addition to ILPs, also SKs (Söderberg et al., 2012). SKs are a satiety signal in various insects (Audsley and Weaver, 2009). Thus far, a clear mode of cooperation of SK and ILPs has not been elucidated. It was shown that knockdown of either neuropeptide affects the transcript levels of the other, suggesting possible feedback regulatory mechanism between the peptides (Söderberg et al., 2012). Recently, it was also shown that SKs influence the ILP level in the haemolymph in the *Tenebrio molitor* beetle, which affects circulating carbohydrate levels (Słocińska et al., 2020).

### Digestion

Brain-originated ILPs directly stimulate digestion in the gut, thus they provide the nutrients used by female *Ae. aegypti* during egg production (Gulia-Nuss et al., 2011). Aedae-ILP3 directly stimulated late phase trypsin-like gene expression in blood-fed females. *In vivo* assays showed that Aedae-ILP3 return digestion to typical level in decapitated females. Moreover, *in vivo* knockdown of IR in *Ae. aegypti* retarded but did not fully excluded late phase trypsin-like gene expression and its activity in the gut as well as ecdysteroid production by ovaries and vitellogenin expression by the adipose tissue. It was also shown that amino acids do not induce the expression of late phase trypsin-like genes in the gut, but they significantly increase the ability of Aedae-ILP3 to direct stimulation of late phase trypsin-like gene expression. This indicates that ILPs released by neurosecretory cells in the brain after blood intake act as main regulators of metabolism, growth and reproduction, synchronizing blood meal digestion and amino acid availability with the production of ecdysteroids by ovaries to maximize vitellogenin expression by the fat body in *Ae. aegypti* (Gulia-Nuss et al., 2011).

In another mosquito species, *A. gambiae*, a mixture of albumin and amino acids (artificial blood) rapidly triggered the transcription of two *ilps* genes: *ilp3* and *ilp4*, in the brains of starved mosquitoes, and the transcripts levels were higher than in mosquitoes fed with sucrose (Arsic and Guerin, 2008; Sharma et al., 2019). In *A. stephensi*, the transcription of *ilps* genes did not change significantly with age or after sugar or blood meal (Marquez et al., 2011; Sharma et al., 2019), which suggests differences in mosquito species.

It was also proven that ablation of IPCs in the brain or reduction of *dilp* gene expression reduced the expression of the target of brain insulin (*tobi*) gene, which encodes a highly conserved α-1,4-glucosidase in the gut and fat body in *D. melanogaster* (Buch et al., 2008). *tobi* expression was dependent on diet, as it is higher after protein ingestion and decreased after sugar meal. After IPCs ablation, diet no longer had any impact: *tobi* was repressed regardless of the nutrients in the meal. This pattern of the opposing regulation of *tobi* by protein and sugar from the diet is reminiscent of the glucagon system in mammals. It was also shown that *tobi* expression was totally inhibited, when the neuroendocrine cells producing AKH, an analogue of glucagon, were ablated. *tobi* is a target of the insulin- and glucagon-like signalling system that reacts in the opposite way to proteins and sugars in the diet (Buch et al., 2008).

### Energy Homeostasis in Trophic Tissues

The major storage forms of carbohydrates in many insects are trehalose present in the haemolymph and glycogen stored in the fat body, but when energy demand increases, insects start to utilize lipids and amino acids. In some insect species, proline might be a main source of energy (Teulier et al., 2016).

The first study investigating the impact of insect ILPs on carbohydrate metabolism included Bommo-ILPs isolated from the silkworm *B. mori* (Nagasawa et al., 1984). It was shown that injection of Bommo-ILP2 into neck-ligated *B. mori* larvae reduced the amount of one of the main haemolymph carbohydrates, trehalose, in a dose-dependent manner (Satake et al., 1997). However, the hypotrehalosemic effect of Bommo-ILP2 may be only larval stage-specific because the injection of this peptide into adult *B. mori* did not result in hypotrehalosemia (Satake et al., 1997; Mizoguchi and Okamoto, 2013). ILPs are thought to regulate circulating trehalose levels. But this activity relates to regulation of the trehalase activity *via* different molecular mechanisms which are species- and developmental-specific (Satake et al., 1997; Broughton et al., 2008; Mizoguchi and Okamoto, 2013). Research by Satake et al. (1997) and Satake et al. (1999) showed that Bommo-ILPs increased trehalase activity in the muscles of *B. mori*, which caused a decrease in the haemolymph trehalose level. Endogenous ILPs were able to activate fat body trehalase *in vitro* through a direct molecular interaction in *T. molitor* (Bounias et al., 1993). In *T. castaneum*, in the regulation of trehalase gene expression by JH, the Trica-ILP2 and ILP signalling pathways are involved. In this beetle, the ILPs role in the controlling of trehalose level is explained *via* regulation of expression of the gene encoding trehalase as well as it might concern influence on trehalose biosynthesis or on trehalose transporter activity (Xu et al., 2013). Lowered level of trehalase in the fat body was observed when the JH level was reduced or because of its action or when *ilp2* gene expression increased. Moreover, decreased JH level and its action lowered the amount of trehalose transporter (TRET) in the gut which increase availability of trehalose in haemolymph (Xu et al., 2013).

It was also shown that Brommo-ILP2 decreased the glycogen content in the fat body and midgut and also increased the amount of active form of glycogen phosphorylase in the fat body, but any effect of Bommo-ILP2 injection on the level of glucose in haemolymph was observed compared to the control (Satake et al., 1997). A recent study showed that Bommo-ILPs facilitate cellular energy production but have no effects on lipid content in the haemolymph and fat body in *B. mori* larvae. Carbohydrates are probably not converted to lipids because their level is not affected by this ILP (Kawabe et al., 2019). Reduction in the trehalose concentration in the haemolymph and glycogen content in some tissues, e.g., muscles, after Bommo-ILP injection can be the result of their increased consumption for cellular energy production (Kawabe et al., 2019). Moreover, particular ILPs affect the trehalose and glucose level in haemolymph in more or less diet-dependent manner. For example, the level of trehalose in haemolymph of *D. melanogaster dilp5* mutants did not change depending on diet whereas in *dilp3* mutants the composition of diet strongly affected this parameter. Similar effects were observed in case of glycogen content in fly bodies. In *dilp2* mutants the glycogen amount in fly body was significantly lower than in wild type, but it did not depend on diet composition what was observed in *dilp3* and *dilp7* mutants (Semaniuk et al., 2018).

Apart from the injection of ILPs into the insect body, the ablation of IPCs in the brain and knockout of genes encoding ILPs were applied in ILP studies. Studies have demonstrated that the ablation of IPCs in the brain of *D. melanogaster* causes elevated carbohydrate levels (trehalose and glucose) in the haemolymph of larvae (Rulifson et al., 2002) and elevated glucose (Broughton et al., 2005) and trehalose levels (Belgacem and Martin, 2006) in adult flies compared to the control. In addition, ablation leads to increased storage of lipids and carbohydrates. Therefore, the ablation of IPCs in the brain, which produces three Drome-ILPs, alters lipid and carbohydrate metabolism and generally causes lowered systemic ILP signalling. This study indicates that one or more Drome-ILPs, Drome-ILP2, Drome-ILP3, or Drome-ILP5, are required to stimulate glucose uptake (Broughton et al., 2005). To identify the functions of particular Drome-ILPs, many studies have been conducted using knockdown methods. Subsequent research suggested that Drome-ILP2 may solely regulate the total trehalose content because knockdown of *dilp2* alone in *D. melanogaster* increases total trehalose correspondingly with IPC ablation but not haemolymph carbohydrate or stored glycogen levels (Broughton et al., 2008). Similar results were obtained by Grönke and Partridge (2010), in their study, and among the mutants for all 7 *dilp* genes in *D. melanogaster*, only *dilp2* mutants had increased whole-body trehalose level, which suggests that it is specifically regulated by Drome-ILP2 (Grönke and Partridge, 2010). Of all the single mutants, only *dilp6* mutants had slightly increased lipid levels compared to the control. Generally, knockout mutations show synergy and compensation of expression between different Drome-ILPs (Grönke and Partridge, 2010). Deletion of *dilps1-5* reduces metabolic activity and decreases triacylglycerol (TAG) levels in larvae and adults of *D. melanogaster*. It also elevates circulating sugar levels but less so than in IPC-ablated insects, which suggests that other signals can also impact that regulation. Deletion of *dilps6-7* does not lead to major metabolic defects. Most likely, *dilp6* is not required for metabolic regulation in *Drosophila* larvae. Interestingly, insects with deletion of *dilps1-5* appear relatively resistant to negative impacts of persistent hyperglycaemia (Zhang et al., 2009). In honey bee larvae, ILPs called Apime-ILP1 and Apime-ILP2 were thought to regulate energy metabolism (Wang et al., 2012); however, another study showed that neither glucose nor trehalose haemolymph concentrations were influenced by these peptides (Wang et al., 2013). In contrast, knockdown of the gene encoding Spoex-ILP1, the first reported ILP gene in the beet armyworm *Spodoptera exigua*, induced a significant, sevenfold increase in haemolymph trehalose levels compared to the control (Kim and Neufeld, 2015).

However, in some insect species, ILPs increase the amount of energy reserves. In the mosquito *Ae. aegypti*, Aedae-ILP3 reduced circulating sugars 6 h after injection, but it also elevated carbohydrate and lipid storage 24 h after injection (Brown et al., 2008). Knockdown of the gene encoding Rhopr-ILP, the first ILP identified in *R. prolixus*, resulted in an increase in carbohydrate and lipid levels in the haemolymph and decreased carbohydrate content in the fat body and leg muscles. These insects exhibited increased lipid content in the fat body and larger lipid droplets compared to the control (Defferrari et al., 2016). Thus, the effects of ILPs on insect metabolism differ between species. Insects feed with long intervals between meals; for example, *R. prolixus* and *Ae. aegypti* may have evolved mechanisms to facilitate their conversion of excess carbohydrates to glycogen or lipid reserves (Kawabe et al., 2019). The effects of ILPs on the level of energy substrates in insect tissues are presented in Table 2 and on Figure 2.

**TABLE 2:** The effects of different ILPs on the level of energy substrates in insect tissues.

**ILP**	**Insect species**	**Method**	**Glucose level**	**Trehalose level**	**Glycogen level**	**Lipid level**	**References**
Bommo-ILPs	*Bombyx mori* larva	injection into neck-ligated	no effect in haemolymph	decrease in haemolymph	decrease in fat body and midgut	–	Satake et al., 1997
Bommo-ILP2	*Bombyx mori* larva	injection into neck-ligated	decrease in haemolymph	decrease in haemolymph	decrease in haemolymph	no effect in fat body and haemolymph	Kawabe et al., 2019
Different ILPs	*Drosophila melanogaster* larva	ablation of IPCs	increase in haemolymph	increase in haemolymph	–	–	Rulifson et al., 2002
Different ILPs	*Drosophila melanogaster* adult	ablation of IPCs	increase in haemolymph	decrease in haemolymph, increase in whole-body extract	increase in whole-body extract	increase in whole-body extract	Broughton et al., 2005
Drome-ILP2	*Drosophila melanogaster* larva and adult	knockdown	no effect in haemolymph	no effect in haemolymph, increase in whole-body extract	no effect	no effect	
Drome-ILP1-5	*Drosophila melanogaster* larva and adult	knockdown	increase in haemolymph	increase in haemolymph	–	decrease in whole body	Zhang et al., 2009
Drome-ILP2	*Drosophila melanogaster* adult	knockout	–	increase in whole body	no effect	no effect	Grönke and Partridge, 2010
Drome-ILP6	*Drosophila melanogaster* adult	knockout	–	–	no effect	increase	
Aedae-ILP3	*Aedes aegypti* adult	injection into neck-ligated	decrease in haemolymph after 6 h	decrease in haemolymph after 6 h	no effect after 6 h, increase after 24 h	no effect after 6 h, increase after 24 h	Brown et al., 2008
Apime-ILP1-2	*Apis mellifera* larva	knockdown	no effect in haemolymph	no effect in haemolymph	–	–	Wang et al., 2012, 2013
Spoex-ILP1	*Spodoptera exigua* larva	knockdown	–	increase in haemolymph	–	–	Kim and Neufeld, 2015
Rhopr-ILP	*Rhodnius prolixus* adult	knockdown	increase in haemolymph, decrease in fat body and leg muscles	increase in haemolymph, decrease in fat body and leg muscles	increase in haemolymph, decrease in fat body and leg muscles	increase in fat body and haemolymph	Defferrari et al., 2016

### Muscle Metabolism

Insulin in vertebrates is considered as an anabolic hormone. This is related to the fact that this hormone participates in the synthesis of carbohydrates, fat, and proteins (Dimitriadis et al., 2011). Additionally, as we mentioned previously, insulin is highly involved in energy homeostasis, *inter alia*, by increasing the rate of glycolysis in muscles by stimulating hexokinase and 6-phosphofructokinase activity and stimulating glycogen synthesis (Dimitriadis et al., 2011). Recent reports concerning the role of ILPs in the regulation of the functioning of insect muscles showed that, similar to vertebrate insulin, these neuropeptides are also important for the regulation of growth and ageing, and the control of energy storage in the muscles (Bai et al., 2013; Bretscher and O’Connor, 2020).

Proper growth of muscles is dependent on ILP signalling. Research by Demontis and Perrimon (2009) and Kim and O’Connor (2021) showed that different components of ILP signalling may be important for the morphological properties (width, thickness, length) and ploidy of insect muscles. The results of these studies showed that the lack of ILP-dependent inhibition of the transcription factor FOXO led to a decrease in the size of *D. melanogaster* muscles (Demontis and Perrimon, 2009). The dependencies of FOXO activity and muscle size are related to the fact that an increase in FOXO levels causes inhibition of the gene encoding Myc. Similar to its vertebrate homologues, Myc is a central regulator of the growth and proliferation of many cell types, including myocytes (Gallant, 2013). Additionally, research carried out by Kim and O’Connor (2021) showed the importance of ILP signalling in the regulation of muscle growth in insects. The authors confirmed that Activin signalling promotes the growth of *D. melanogaster* muscles by positive regulation of the insulin receptor IR/TORC1 pathway and the level of Myosin heavy chain (Mhc) by increasing pdk1 and akt1 expression, genes encoding phosphoinositide-dependent kinase 1 and Akt kinase (Gallant, 2013). Moreover, Activin participates not only in controlling insect muscle growth but also in the functional ageing of this tissue. Research by Bai et al. (2013) showed that Activin is a direct, downstream target of ILPs/FOXO signalling within *Drosophila* muscles and may non-autonomously regulate lifespan. It should be mentioned that elevated expression of the gene encoding FOXO in muscles causes maintenance of protein homeostasis and delays the ageing-related decline in muscle activity (Bai et al., 2013).

Insulin-like peptides are important not only for muscle growth but also for muscle functioning. Research by Gorczyca et al. (1993) showed that IRs in *D. melanogaster* are located around synaptic boutons near the nerve branch point at each fibre of body-wall muscles. Additionally, insulin-like immunoreactivity was found in some body wall muscles (Gorczyca et al., 1993). These results are partially confirmed by Veenstra et al. (2008) because *ilp3* mRNA was found in muscle cells of the midgut.

Despite the participation of ILP signalling in insect muscle growth and functioning, regulation of the availability of energy substrates stored in insect muscles by ILPs is no less important (Bretscher and O’Connor, 2020). The main energy substrate accumulated in insect muscles is glycogen. Similar to vertebrates, glycogen synthesis in insects is controlled in the muscles by ILP signalling (Yamada et al., 2018; Bretscher and O’Connor, 2020). However, ILPs participate not only in the regulation of glycogen synthesis but also in energy liberation by breaking down glycogen to glucose. Research by Post et al. (2018) showed that glycogen phosphorylase (GlyP), which is involved in this process, is negatively regulated by ILP signalling, specifically by Drome-ILP-2. Additionally, it should be mentioned that GlyP activation is related to the action of AKH, which once again presents strong relationships between these two neuropeptides (Post et al., 2018; Ahmad et al., 2020). Glycogen breakdown not only depends on GlyP activity but can also occur through autophagy. In the fat body, starvation-induced autophagy requires inhibition of the TOR pathway, which is inextricably linked with ILP signalling (Kannan and Fridell, 2013; Zirin et al., 2013). Due to some resemblances between the regulatory roles of ILPs in the fat body and muscles, we can assume that similar dependencies may also be present in insect muscles, such as a reduction in glycogen content in leg muscles of *B. mori* by Bommo-ILP (Kawabe et al., 2019) or reduced content of carbohydrates in *R. prolixus* leg muscles after knockdown of genes encoding Rhopr-ILPs (Defferrari et al., 2016) and increased trehalase activity in the muscles of *B. mori*. However, the results of recent research concerning the role of ILPs in energy homeostasis in insect muscles are not consistent and strongly depend on the model organism used, as mentioned before.

Interestingly, insect ILP signalling is also involved in lipid storage in the muscles. Research conducted on *D. melanogaster* by Zhao and Karpac (2017) showed that an increase in ILP signalling in the muscles causes the presence of TAG in this tissue. Moreover, similar to the storage of carbohydrates, this process involves the FOXO transcription factor (Zhao and Karpac, 2017).

### Reproduction

It is well known that reproductive processes are energetically demanding, so it is therefore no surprise that they undergo complex pathways related to lipid and carbohydrate availability, reallocation, and metabolism (Fullbright et al., 1997; Nässel and Vanden Broeck, 2016; Leyria et al., 2021a). It was shown that ILPs and ILP signalling are necessary for correct functioning of both the female and male reproductive systems. In adult insects, disturbances in ILP levels might disrupt direct reproduction processes as well as hormone synthesis and release (Koyama et al., 2008). For example, mutations in insect ILP signalling alter JH synthesis in the CA, probably as an effect of the reduction of 3-hydroxy-3-methylglutaryl CoA reductase (HMG CoA reductase), a key enzyme in cholesterol biosynthesis necessary for JH formation (Tatar et al., 2001; Tu et al., 2005; Belgacem and Martin, 2006). Another relationship between ILPs and reproduction is ovarian ecdysteroid synthesis, which has been investigated in the mosquito (Nässel and Vanden Broeck, 2016). It was found that in adult female mosquitoes after a blood meal goes to ILPs releasing, what causes induction of ovarian ecdysteroidogenesis probably as an effect of binding of endogenous Aedae-ILP3 to the mosquito IR localized in cell membranes of follicle and nurse cells (Riehle and Brown, 2002; Brown et al., 2008; Wen et al., 2010). Additionally, in *R. prolixus*, a positive receptor signal was found in the tropharium, specifically in the cell membranes of nurse and follicle cells which surrounds oocytes in vitellogenic stage (Leyria et al., 2021b). Tu et al. (2002) also pointed out that in *Drosophila*, ecdysone is synthesized by follicle cells, and its secretion remains under the control of IR signalling.

With regard to reproduction processes, it was also shown that they undergo insulin pathway control. These neuropeptides, along with TOR, play crucial roles in acting as nutritional sensors (Badisco et al., 2013). This was confirmed by the results of Leyria et al. (2021b), who demonstrated that *R. prolixus* ILPs/ToR signalling in fat body tissue as well as in ovaries transduces the signal *via* Akt (protein kinase B) and is active only in fed insects. When considering dietary changes, it was shown that proper functioning of *Drosophila* ILP signalling is necessary to regulate egg production. Changes in that process might occur at different stages. First, germline cell division in *Drosophila* appears to be controlled in direct manner by central nervous system derived insulin (LaFever and Drummond-Barbosa, 2005). LaFever and Drummond-Barbosa (2005) showed that on a protein-poor diet, rates of division and development are decreased, and vitellogenesis processes are blocked. Moreover, it was shown that *Drosophila* ovarian cells require an undisturbed insulin pathway to properly function in cell proliferation and apoptosis cycling to enter vitellogenesis (Drummond-Barbosa and Spradling, 2001). The secretion of ILPs and their further activity are glucose-mediated, and it is possible that a disturbance in ILP signalling leads to deficiency of yolk protein absorbtion, which might explain the sterility (inhibited egg production as an effect of impaired development of the primary oocytes and termination of oocyte growth before relocation of the follicles despite the well-formed egg chambers) of *Drosophila* Chico mutants, as well as *T. casteneum* IR, Chico or TOR knockdown females (Drummond-Barbosa and Spradling, 2001; Richard et al., 2005; Parthasarathy and Palli, 2011). *D. melanogaster* female IR mutant ovaries remain stopped at the pre-stage of vitellogenesis but adding a methoprene (juvenile hormone analogue) retrieve vitellogenesis (Wu and Brown, 2006). In contrast, in late vitellogenic follicles of *R. prolixus*, no IR signal was found, probably since the oocytes internalized the nutrients needed for egg formation, which might prove that ILPs are engaged in the maintenance and increase of vitellogenic follicles (Leyria et al., 2021b). This is also supported by results from Silva-Oliveira et al. (2021), in which it was shown that IR-deficient *R. prolixus* females possess smaller ovaries and oviposition is reduced. Additionally, Al Baki et al. (2019) showed that in the reproductive period of *Maruca vitrata*, ILPs exhibited increased levels of gene expression. Observation of the terminal region of ovarioles shows that after treatment with dsRNAs specific to Marvi-ILP1 or Marvi-ILP2, the number of diving cells was decreased (Al Baki et al., 2019). This proves that in *M. vitrata*, ILPs also play crucial roles in mediating cell proliferation and triggering vitellogenesis (Al Baki et al., 2019). Similar effects were observed in *Ae. aegypti* (Riehle et al., 2006; Gulia-Nuss et al., 2011). One hour after blood feeding, the endocrine cascade starts, and ILPs and different neuroendocrine agents activate ovaries to secrete ecdysteroid hormones (ECDs) into the haemolymph, which is regarded as a first step for egg growth and development, as an effect of ECD signalling in the activation of vitellogenesis (Riehle et al., 2006; Wu and Brown, 2006; Gulia-Nuss et al., 2011).

As it has become clear that ILP signalling pathways are key factors in reproductive physiology, no wonder that ILP signalling is one of the basic mechanisms that controls diapause (Badisco et al., 2013). Reduced juvenile hormone level, observed in diapausing insects during diapause is potentially the eliciting factor and presumably the result of reduced ILP signalling (Tatar et al., 2001). There are also reports that FOXO plays a key role, which remains under the control of ILPs, since knockdown of this molecule inhibited *Culex pipiens* from entering diapause in addition to the data that FOXO seems to be present in the fat body of mosquitoes during diapause in high levels (Sim and Denlinger, 2008).

The lack of literature data on ILP signalling function in female insects in relation to male reproductive physiology shows that little attention has been conferred upon this issue thus far. Despite less documented data, insect ILP signalling has been studied as a coordinator of several aspects of male reproductive physiology when considered together with nutritional state. ILP signalling regulates spermatogenesis in male *Drosophila* insects, directly influencing the maintenance and proliferation of germline stem cells in testes (Ueishi et al., 2009; McLeod et al., 2010). According to Masly et al. (2011), ILP signalling also affects the growth of both male external genitalia, as well as horns, used to compete with rivals, in horn beetles such as *Onthophagus nigriventris* (Emlen et al., 2012; Lavine et al., 2013). Similar observations were made on *Trypoxylus dichotomus* and manifested in a 16% reduction in horn length after IR knockdown (Emlen et al., 2012).

There are reports that larval growth also operates under ILP coordination (Nagasawa et al., 1984; Emlen et al., 2012). The expression level of *Bombyx* ILP signalling pathway genes (such as InR, IRS or PDK) in fat body tissue was upregulated during moulting and pupation (Liu et al., 2010). Additionally, in *Drosophila*, Drome-ILP6 is produced in larger amounts during metamorphosis (Slaidina et al., 2009). Furthermore, it was shown that *M. vitrata* larvae treated with Marvi-ILP1 and Marvi-ILP2 RNAi exhibited significant growth retardation, which in some cases manifested with higher mortality (Al Baki et al., 2019).

### Immune Activity

Insulin-like peptides, similar to other insect neuropeptides, probably exert direct immunotropic activities on immune-related cells. This supposition is supported, for example, by research conducted on *Ae. aegypti*. The results obtained by Castillo et al. (2011) showed that the expression of the gene encoding IR was found in phagocytic granulocytes and oenocytoids. Also, ILP singling could be involved in the regulation of antimicrobial peptides (AMPs) genes expression (Becker et al., 2010; McCormack et al., 2016; Urbański and Rosiński, 2018). In addition, recent results strongly suggest that ILP signalling is strictly connected with immune-related pathways and participates in the regulation of metabolic changes related to the activation of immune mechanisms (Dolezal et al., 2019).

Generally, activation of immune cells leads to suppression of systematic metabolism (Dolezal et al., 2019). Additionally, some elements switch their functions. A perfect example is apolipophorin III, which participates in the pathogen recognition process and increases lysozyme activity, but during the stress response apolipophorin III, is mainly involved in lipid transport (Adamo et al., 2008; Zdybicka-Barabas et al., 2013). Many of these metabolic changes are related to ILPs, both at the molecular level by blocking ILP signalling and at the cellular level by affecting ILP release (Dolezal et al., 2019). Interestingly, recent research clearly indicates that activation of all the main immune-related pathways (Toll, Imd, JAK/STAT) elicits effects associated with ILP signalling.

The Toll pathway is crucial for the response of insect organisms to infection by various pathogens, including bacteria, fungi, and viruses (Vigneron et al., 2019). Activation of this pathway causes AMP synthesis and modulates the activity of the cellular response (Johnston et al., 2014; Shafeeq et al., 2018). Research by DiAngelo et al. (2009) showed that activation of the Toll pathway in *D. melanogaster* may also suppress ILP signalling in the fat body, which results in a reduction in nutrient storage. These results are supported by Suzawa et al. (2019), which demonstrated that activation of the Toll receptor suppresses animal growth. The observed effect of Toll activation is related to the reduction in the level of circulating Drome-ILP6. Interestingly, restoring the expression of *ilp* in the fat body upon activation of the Toll pathway rescued the growth of tested fruit flies (Suzawa et al., 2019). It should also be highlighted that activation of the Toll pathway depends on the PGRP-SA receptor (peptidoglycan-recognition protein SA) and cytokine Spätzle. Transcripts of genes encoding these proteins are also found in insect haemocytes; for this reason, we can assume that haemocytes play a role in the activation of humoral immunity associated with a metabolic switch (Dolezal et al., 2019).

The second, but no less important, set of molecular pathways involved in the regulation of immune system functions and ILP signalling is the Imd (immunodeficiency) pathway (Zhai et al., 2018). Imd signalling plays a pivotal role in insect defence against microorganisms, especially Gram-negative bacteria (Kleino and Silverman, 2014). Interestingly, despite dependencies between the functioning of the Toll and Imd pathways, activation of the Imd pathway does not antagonize ILP signalling (DiAngelo et al., 2009). However, Eiger (an orthologue of tumour necrosis factor α, TNF-α), one of the cytokines important for activation of the Imd pathway, may also modulate ILP signalling. A recent study showed that Eiger can bind to the Grindewald receptor in IPCs, which may result in the inhibition of *D. melanogaster* growth by reducing the expression of genes encoding ILPs (Agrawal et al., 2016).

Similar to the Toll and Imd pathways, JAK/STAT activation modulates the activity of immune mechanisms and insect metabolism *via* ILP signalling. JAK/STAT is a highly conserved molecular pathway in insects that participates in the regulation of immune system activity as well as cell growth, differentiation, and apoptosis (Bang, 2019). JAK-STAT signalling is activated by the Unpaired 3 (Upd3) cytokine. Recent studies have shown that the appearance of this cytokine may have some implications not only for immune system functioning but also for muscle metabolism (Bretscher and O’Connor, 2020; Kierdorf et al., 2020). Releasing Upd3 from haemocytes during infection could reduce ILP sensitivity in muscles by activating JAK-STAT. This results in inhibition of glucose consumption by muscles and the redirection of available energy to haemocytes, which is required during defence against pathogen infection (Gallant, 2013; Zhao and Karpac, 2017). The results obtained by Lourido et al. (2021) also support supposition about the close relationships between JAK/STAT and ILPs in the regulation of insect metabolism. These authors showed that loss of the Domless receptor (part of JAK/STAT pathway) in the fat body of *D. melanogaster* reverses hyperglycaemia and increases the expression level of the insulin resistance marker nlaz in larvae on a high sugar diet.

## Summary

Analysis of data concerning the role of ILPs in the regulation of insect physiology shows that this peptide family is one of the crucial groups of peptide hormones that control insect life, which is in line with situations occurring in mammals or more generally in vertebrates. The comprehensive role of ILPs results from their metabotropic activity. They regulate the insect’s nutritional status at various levels, and the intake and utilization of nutrients underlie all other life processes (Figure 2). Their multidirectional activity produces outcomes at several levels: (1) multiplicity of ILP family members, (2) ubiquity of production by various cells, (3) commonality of prevalence of IR receptors in different tissues, and (4) interplay of ILP signalling pathways with signalling pathways of other hormonal and nonhormonal factors. Because of the above, as well as the fact that ILPs affect cells/tissues/organs both in a direct and indirect manner, studies concerning the role of these peptides, their mode of action, and the mechanism regulating their production and secretion are not simple, and the obtained data are not easy to interpret. Moreover, it has to be borne in mind that the interpretations and comparison of different results about ILPs activity is all the more hard to interpret because of many various techniques and approaches used in research on ILPs. Even, if studies concern the same aspect, the used techniques do not allow for direct comparison or interpretation. Thus, although knowledge about ILPs increases from year to year, many aspects of their activity remain unclear and unknown. On the other hand, one of the most ancient regulatory systems is widespread in the animal kingdom, and the high similarity of insect and mammalian ILP signalling systems allows the use of insects as models for many human disorders and illnesses, e.g., obesity or diabetes.

As was mentioned above, ILPs are involved in regulation of almost all life processes in insects and their activity and mode of action is highly complexed and, in many points, crosses with other hormonal and non-hormonal systems regulating insect physiology. Because of that, still many of their activities are unclear and remain unknown. Nevertheless, that gives possibility for the next research with many perspectives. All the time the number of studies with knockdown of genes encoding ILPs (with single or multigene knockdown) increases. But still, the research about cross-talks with other factors is scanty. For example, simultaneous knockdown of *ilps* gene and genes encoding other hormonal factors like AKH, sNPFs or SKs. Also studies with application of ILPs together with other factors are not numerous. This research approach with e.g., pre-treating with SKs or sNPFs might show which signal dominates over another and “is more important”. Moreover, the role of nervous system in regulation of ILPs synthesis or secretion is not well explored. It is known that muscarinic receptors are involved in secretion of Bommo-ILPs, but how they interplay with other factors? Since the ILPs are a crucial controllers of metabolism, it is interesting what is their role in response to stress, e.g., cold stress or whether and how signalization *via* ILPs regulates mitochondria activity.

Despite that insulin-like peptides are one of the most explored group of peptide hormones in insects, and not only in insect, there are also still a lot of to do and discover.

## Author Contributions

SC and JP-B contributed conception of the manuscript. SC, KW-N, MW, PM, AU, and JP-B wrote and edited the manuscript. SC coordinated the preparation of manuscript. All authors contributed to the article and approved the submitted version.

## Conflict of Interest

AU is employed by the company HiProMine S.A. The remaining authors declare that the research was conducted in the absence of any commercial or financial relationships that could be construed as a potential conflict of interest.
